# Unveiling Safety Concerns in Brazilian Artisanal Cheeses: A Call for Enhanced Ripening Protocols and Microbiological Assessments

**DOI:** 10.3390/foods13111644

**Published:** 2024-05-24

**Authors:** Tatiane Mendonça Nogueira Carneiro de Albuquerque, Gabriela Zampieri Campos, Loredana d’Ovidio, Uelinton Manoel Pinto, Paulo José do Amaral Sobral, Julia Arantes Galvão

**Affiliations:** 1 Department of Veterinary Medicine, Federal University of Paraná, Curitiba 80060-000, Brazil; nogueira.tm@ufpr.br; 2 Department of Food Sciences and Experimental Nutrition, Faculty of Pharmaceutical Sciences, University of São Paulo, São Paulo 05508-220, Brazil; gabrielazcampos@gmail.com (G.Z.C.); loredanadovidio@usp.br (L.d.); uelintonpinto@usp.br (U.M.P.); 3 Food Research Center (FoRC), University of São Paulo, São Paulo 05508-220, Brazil; pjsobral@usp.br; 4 Faculty of Animal Science and Food Engineering, University of São Paulo, Pirassununga 13635-900, Brazil

**Keywords:** microbiological indicators, cheese maturation, foodborne pathogens, food safety, traditional methods, zoonotic pathogens

## Abstract

Brazilian artisanal cheeses have recently gained significant commercial prominence and consumer favor, primarily due to their distinctive sensory attributes and cultural and historical appeal. Many of these cheeses are made with raw milk and undergo a relatively short ripening period, sometimes ranging from 4 to 8 days, though it is usually shorter than the period stated by law. Moreover, there is insufficient evidence regarding the efficacy of a short ripening period in reducing certain zoonotic foodborne pathogens, such as *Brucella* spp., *Coxiella burnetiid*, and *Mycobacterium bovis* (as part of the *Mycobacterium tuberculosis* complex). Additionally, a literature analysis revealed that the usual ripening conditions of Brazilian artisanal cheeses made with raw milk may be inefficient in reducing the levels of some hazardous bacterial, including *Brucella* spp., *Listeria monocytogenes*, coagulase-positive *Staphylococcus*, *Salmonella*, and *Coxiella burnetti*, to the acceptable limits established by law, thus failing to ensure product safety for all cheese types. Moreover, the assessment of the microbiological safety for this type of cheese should be broader and should also consider zoonotic pathogens commonly found in bovine herds. Finally, a standardized protocol for evaluating the effectiveness of cheese ripening must be established by considering its peculiarities.

## 1. Introduction

According to the Dairy Industry Union of *Minas Gerais* state, Brazil produced 1.2 million tons of cheese in 2020. Approximately, 70 types of cheese are manufactured in Brazil, with mozzarella, *prato* cheese, parmesan, and *Minas* fresh cheese constituting around 60% of the total production [1,2]. However, artisanal cheeses have recently gained significant prominence due to their distinctive quality [3]. In accordance with Brazilian Federal Law 13,860/2019 [4], artisanal cheeses are those produced using traditional methods. They are linked to and valued for their geographical or cultural origin, and they follow a specific manufacturing protocol that applies good manufacturing and agricultural practices.

The manufacturing process of each kind of artisanal cheese is unique and may differ between regions or even within regions depending on the individual producer [5]. Milk coagulation occurs by adding coagulant agents, such as commercial rennet (chymosin) or organic acids. Endogenous or industrial starter cultures may also be added at this step [6]. Ripening takes place over varying lengths of time and under different environmental conditions for each type of artisanal cheese [3,5,7].

A type of endogenous culture called *pingo* is used in the production of the Queijo *Minas artesanal* (QMA—Artisanal Minas Cheese) and *Caipira* cheese. *Pingo* is a natural starter culture composed of fermented whey from the previous day’s batch, containing lactic acid bacteria (LAB)—such as those from the genera *Lactobacillus*, *Lactococcus*, *Streptococcus*, and others [8]. The most abundant bacterial species in the pingo from *Canastra* cheese are *Lactococcus lactis*, *Streptococcus thermophilus*, *Streptococcus infantarius*, *Streptococcus salivarius*, and *Corynebacterium variabile* [9]. Additionally, a portion of the cheese that is grated after the ripening process and known as *rala* could be considered as a substitute for *pingo*. It is used to reduce cheese microbial contamination and improve its consistency [10,11]. Both *pingo* and *rala* are used to give a unique and peculiar flavor, aroma, and texture to each type of traditional cheese.

Many artisanal cheese types, such as *Arupiara*, *Cariri*, *Caipira*, *Colonial*, QMA, and *Serrano*, which are made from raw milk, do not undergo thermal processing during production [5]. Therefore, a later step in the process, such as ripening, is necessary to reduce any raw milk contamination and to guarantee the final product’s safety [12,13,14,15]. Brazilian law has determined that ripening cannot be shorter than 60 days unless there is scientific evidence proving that reducing this period does not jeopardize cheese quality and safety [16]. Many of the approved ripening times, particularly for QMA, are between 14 and 22 days, and they are in accordance with a few studies that have showed that this period of time is sufficient to reduce the levels of indicator organisms such as coliforms, *Escherichia coli*, and coagulase-positive *Staphylococci* (CPS) [3].

Various authors have determined the impact of cheese microbiota during ripening and storage. They have shown that regional origin, manufacturer, or even the type of making process can interfere in the product’s sensorial, physical–chemical, and microbiological characteristics. Furthermore, artisanal cheeses of the same type produced in the same region, supposedly using the same manufacturing protocol, have distinct microbiota and sensorial characteristics from each other due to geoclimatic properties (*terroir*) in addition to each producer’s specific environment and manufacturing distinctions [3,5,7,12,14,17,18].

Brazilian cheeses have stood out in international festivals such as 5th Mondial du Fromage in France [19,20], the 3rd *Mundial do Queijo do Brasil* in São Paulo [21], and the World Cheese Awards in Wales [22]. However, national legislation regarding the marketing of these products is controversial. As they are artisanal products and do not have a federal inspection seal, they cannot be sold outside the municipality or state where they are produced [23,24,25]. Thus, to overcome this obstacle, the *Selo Arte* (Artisanal Seal) and *Selo Queijo Artesanal* (Artisanal Cheese Seal) were created [26,27]. The first seal can be applied to animal-derived artisanal products, including cheeses, bee-derived products, fish and meat-cured products, etc., and the second seal is specific to cheese. In fact, the Artisan Cheese Seal is a certificate that confirms that artisanal cheeses have been produced using traditional methods with a connection to and valorization of the local territory [27]. Agro-industries that follow the requirements recommended in the legislation and obtain any of these seals can now sell their products throughout the national territory, even when only subjected to municipal or state inspection systems [27].

Considering the global consumer demand for artisanal cheese and their legal and informal trade, the purpose of this manuscript is to present a critical literature review about the behavior of pathogenic bacteria during the ripening process. Moreover, this review aims to verify whether the available literature confirms if the traditional ripening conditions are indeed effective in ensuring the safety of the cheeses in accordance with the established microbiological standards.

In the available literature at the time of research (May 2024), a total of 51 articles published between 2003 and 2024 were found.

The literature search revealed a limited number of studies on ripened cheeses made with the milk from species other than cows. Thus, this review focuses on ripened cheeses made with raw cow’s milk.

To facilitate the analysis of the searched material, the data were organized into related topics, as presented below.

### 1.1. Bacterial Contamination in Brazilian Artisanal Cheeses

Most Brazilian artisanal cheeses are made with raw milk and do not undergo a heat treatment capable of reducing microbial contamination. Satisfactory microbiological conditions for milk, and *pingo* or *rala*, are essential for a low population of undesirable microorganisms in the cheese, albeit as long as the adequate sanitary status of the herd for bovine brucellosis and tuberculosis is maintained. Moreover, inadequate milking practices may result in an increased microbial load of indicator or pathogenic microorganisms in raw milk. Likewise, the quality control of water used in production is also critical. Therefore, the detection of high levels of hygienic indicator bacteria (such as coliforms, *Escherichia coli*, and coagulase-positive *Staphylococcus*) in the cheese may indicate flawed hygienic conditions throughout the manufacturing process [28].

Cheeses sold in Brazil must comply with the microbiological standards set by Brazilian Federal Ordinance n° 146 [29]. This standard stipulates that, for medium-moisture cheeses (up to 46%) such as ripened cheese, maximum populations of 3.7 log CFU/g of total coliforms, 2.7 log CFU/g of thermotolerant coliforms, and 3 log CFU/g of coagulase-positive *Staphylococcus* (CPS) are tolerated. *Salmonella* sp. and *L. monocytogenes* must be absent in 25 g of sample [29]. Even though the Minas Gerais state Decree n° 42,645 [30] and Decree 44,864 [31] set standards specifically for QMA, they are the same as those set in the federal rule. Therefore, in this review, the standards set by Ordinance n° 146 will be used for the analysis of the results of the assessed articles.

The literature review showed that some of the most popular ripened artisanal cheeses produced in Brazil do not comply with the microbiological standards established by Brazilian Federal Ordinance n° 146 [29] (Table 1).

The staphylococci contamination of ripened cheese, as described earlier [43,44], most likely happens due to the use of milk from animals with clinical or subclinical mastitis. This emphasizes that the control of herd health is essential to obtain good quality cheeses [42]. Furthermore, studies have shown that food handlers play an important role in the contamination of products, acting as source of bacteria (such as *Staphylococcus* spp.) for cheeses. This type of contamination can be avoided by adopting good agricultural, milking, and manufacturing practices [43].

In this context, some authors [10,28,34] have reported CPS levels greater than the legal limit (3 log CFU/g) in QMA cheese. Moreover, CPS genes responsible for producing toxins that cause gastroenteritis in susceptible individuals were detected in some of these cheeses [28,45,46], as well as *E. coli* toxin genes [36].

In addition to *Staphylococcus*, another relevant pathogen is *Salmonella.* This kind of fecal-origin contamination occurs due to inadequate agricultural and industrial practices and due to the fact that the microorganism thrives at improper storage temperatures. Degenhardt et al. [47], observed that *S. enteritidis*, which was intentionally added in *Colonial* cheese samples (3.0 Log CFU/L), was able to survive for at least 28 days of ripening but was no longer detected at 35 days. Allaion et al. [34] demonstrated that most QMA that are positive for *Salmonella* sp. also present high counts of total coliforms, *E. coli*, and CPS. As a result, the authors suggested a correspondence between deficient hygiene during cheese making and a higher risk of *Salmonella* sp. detection. The results found by Delamare et al. [42], Hipólito et al. [40], and Rezende et al. [35] support this hypothesis.

However, in most studies, *Salmonella* sp. [12,15,18,28,33,35,36,48,49] and *L. monocytogenes* [10,13,18,33,36,40,48,49] were absent in the various kinds of artisanal cheese surveyed. It is worth noting, though, that Allaion et al. [34] detected 8 positive samples (out of 100) for *Salmonella* by PCR, but only one was confirmed by the culture-dependent method.

An important highlight is that the time-temperature binomial for milk pasteurization is based on the inactivation of *C. burnetii*, which is one of the most resilient and thermoresistant pathogens found in this product. It causes the Q fever [50], an important zoonosis, and it is mainly transmitted to humans by aerosol inhalation but can also be transmitted by the consumption of contaminated unpasteurized milk and dairy products [32,39].

The pathogen *C. burnetii* can resist the ripening process of cheeses made with raw milk for up to eight months despite low pH (4.96–5.41) and low a_w_ (0.907–0.953) [51]. However, it is not a target microorganism in Brazilian health surveillance services, and this is in addition to it not being evaluated in studies that have supported the creation of the current legal standards.

Furthermore, Rozental et al. [32] and Nascimento et al. [39], respectively, detected *Coxiella burnetii* DNA in samples of *Serro* and *Cerrado* cheeses. Although Nascimento et al. [39] took samples from products sold informally, i.e., without official inspection, their results found less frequent contamination than those from Rozental et al. [32], who took samples from registered products that underwent inspection from regional authorities. Rozental et al. [32] estimated that 1.62 ton/day of ready-to-consume Serro cheese may be contaminated by this bacterium, representing almost 10% of the overall production for this cheese.

Moreover, *Brucella abortus* and *Mycobacterium bovis* are endemic in many Brazilian regions, and they are the cause of important cases of zoonosis [52]. Miyashiro et al. [53] used PCR analysis, and they found the DNA of *Brucella abortus* in 15.68% of samples of ripened *Minas* cheese that were informally marketed in the states of *São Paulo* and *Minas Gerais*. Of those, 18.92% were classified as field strains—in other words, strains derived from infected animals—indicating the presence of brucellosis in bovine herds.

Likewise, Silva et al. [54] found that 31% of *Serro* cheese samples were positive for the presence of field strains of *Brucella* sp., even though they came from serologically negative herds. Silva et al. [55] found that, in 1.8% of the samples, *B. abortus* biovar 2 field strains were recovered by cultivation. These latter two studies highlight that their samples were taken from cheeses ripened for periods ranging from 4 to 8 days, and they were already marketed after an official certification that the herd is free of brucellosis. This indicates that *Serro* cheese may be a source of *Brucella* contamination for humans even from a free-brucellosis-certified herd. In contrast, Andretta et al. [10] did not detect this agent in ready-to-eat *Serro* cheese.

Regarding *M. bovis*, there are scarce studies [10,56] in the literature that have assessed the presence of this microorganism in ripened Brazilian artisanal cheeses. However, Moriconi et al. [56] did not find this pathogen in *Minas* cheese samples acquired from open fairs in *São Paulo* city, Brazil. Additionally, Andretta et al. [10] did not detect this bacterium in *Serro* cheese.

Variations in results were observed among the authors during the analysis of the literature on cheese bacteria. These variations can be attributed to differences in the types of cheese analyzed, production regions, and individual manufacturers. Additionally, the choice of analysis methods can significantly influence the outcomes. A significant proportion of articles, especially older ones, relied on traditional bacterial plate cultivation practices. In contrast, more recent studies have increasingly employed culture-independent methods, such as 16S rRNA gene sequencing. Generally, the use of different techniques results in the observation of distinct bacterial species. However, there is a limited availability of studies using Next Generation Sequencing techniques for an objective comparison between methods.

The Regional Ordinance IMA N° 1969 [57], which is in regard to QMA production techniques, states that, for *Serro* cheese, the ripening period must be no shorter than 17 days. However, it is common that some cheeses are sold after being ripened for only 4 to 8 days. In those cases, the cheese pH does not decrease as much as needed to inactivate *Brucella*. Therefore, the consumption of cheese with a low ripening time represents a great health hazard [54,55].

Moreover, the regulations require that raw milk cheeses ripened under 60 days must meet specific criteria, including a property certification that it is free of tuberculosis and brucellosis and that it conducted mastitis control, milk analysis, and adherence to good practices. Specifically, Law N° 13,860 [4], regarding artisanal cheese production and marketing, dictates that rural properties must be certified as free of brucellosis and tuberculosis, as determined by the National Control and Eradication of Brucellosis and Tuberculosis Program (PNCEBT), to produce artisanal cheeses made with raw milk.

All the same, despite the fact that *Brucella* DNA detection does not implicate the presence of viable bacteria in the cheese, it does suggest that there is still pathogen circulation in the milk-producing herds. Thus, future studies should consider assessing *Brucella* sp. field strain viability in products. Furthermore, one should also consider that the number of producers that are not registered in official inspection services is a great deal higher than the number of those that undergo this control [3].

Until 2018, in Brazil, there were 1932 and 1988 farms certified free of brucellosis and tuberculosis, respectively [58]. This corresponded to 0.04% of the total number of national properties when considering that there are approximately 5 million registered rural establishments in the country [59]. Thus, the guarantee that most properties are free of brucellosis and tuberculosis is far from being a reality.

Legal microbiological standards established for dairy products only consider the evaluation of total coliforms, thermotolerant coliforms, coagulase-positive staphylococci, *Salmonella* sp., and *L. monocytogenes* [29]. However, the findings from the aforementioned studies show that more comprehensive criteria should be instituted for raw artisanal cheese since contamination by *Brucella*, *C. burnetiid*, and staphylococcal toxin were found in some studies. It is important to note that most studies in Brazil focusing on the microbiological safety of raw milk cheeses do not assess the presence of these specific zoonotic pathogenic agents. This is partly because the current legislation already requires properties to be certified as free of brucellosis and tuberculosis before producing such cheeses. However, 99% of Brazilian properties cannot be certified free of these diseases [58,59]. It is worth mentioning that Minas Gerais state, i.e., the major producer of milk and this type of cheese in the whole country [60], has just 40 certified properties free of brucellosis and tuberculosis [61].

### 1.2. The Ripening and Safety of Brazilian Artisanal Cheeses

Although Brazilian federal law allows for the manufacturing and marketing of cheese made with raw milk, it also determines that it should be ripened for a period of at least 60 days, at temperatures exceeding 5 °C, and that it allows for a reduction in ripening time if a scientific study demonstrates that a shorter period is sufficient to ensure the safety of the product [29]. This temperature × time binomial was defined considering the elimination of specific zoonotic pathogens (*Brucella*, *Mycobacterium*, and *Coxiella burnetii*). On the other hand, producing states have autonomy to regulate regionalized products. Regional Decree N° 1238 of 2017 [62], which is about the production and marketing of *Serrano* cheese in the *Santa Catarina* state, determines that the ripening period must last at least 60 days at room temperature. Conversely, for the same type of cheese made in *Rio Grande do Sul* state, Normative Instruction SEAGRI N° 7 of 2014 [63] states that the minimum ripening time is 60 days at temperatures higher than 5 °C. Regional Ordinance IMA N° 1969 [57], which was altered by Ordinance IMA N° 2051 [64] and is about QMA production, mandates a ripening period of at least 17 days for *Serro* cheese and 14 days for *Araxá*, *Canastra*, and *Serra do Salitre* cheese. It also specifies that ripening must occur at room temperature or under controlled temperatures between 12 to 18 °C, and it can only be refrigerated to lower temperatures after the end of the ripening period.

However, these conditions have been contested by specialists and, particularly, by cheese producers. The extended ripening period leads to physical–chemical, structural, and flavor changes, thus affecting consumer acceptance of the product [65]. Consequently, regulations have been updated to stipulate that the ripening process may be shorter as long as there are technical and scientific evidence demonstrating that the product maintains its quality and harmlessness [16,29]. It is important to note that the scientific studies used to approve shorter ripening periods did not evaluate specific zoonotic pathogens and other pathogens (like *Escherichia coli* O157:H7), thus leaving a gap in product safety assurance.

The majority of the reviewed studies assessed *Serro* cheese samples [14,28,66]. The time to achieve the adequate conditions, as established by law, ranged from 17 to 60 days. However, only Martins et al. [14] demonstrated that 17 days at room temperature were sufficient to achieve microbiological safety. In contrast, Mata et al. [66] determined that artificially contaminated cheese with *L. monocytogenes* supported the viability of the pathogen for 60 days of ripening or more.

Regarding *Canastra* cheese, the minimum ripening time to ensure it becomes a safe product to consume ranged from 14 to 64 days [12,18] depending on the season. It is worth noting that a shorter time (14 days) was achieved for samples collected from artisanal cheese-producing farms registered in the state inspection service and by adhering to good manufacturing practices. Otherwise, even after 22 days of ripening, there was a high number of non-compliant samples, particularly for CPS counts [12].

In their study, Lima et al. [67] observed that the ripening time of *Serra do Salitre* cheese needed to exceed 45 days to achieve a safe product in accordance with the Brazilian Regulatory Standards [29].

Firmo et al. [37] observed that 59% of the producers assessed in their study did not comply with the recommended minimum ripening period; thus, 40.2% of all the QMA samples were microbiologically ineligible for consumption. Cheese producers that followed the minimum ripening time had less than 25% of unsuitable samples. In contrast, cheese samples from those that did not respect the minimum ripening time had more than 50% cases of non-compliance. Other than that, for *Serro* cheese specifically, up to 61% of the samples that underwent a shorter than recommended ripening process were deemed unsuitable for consumption.

Concerning the *Serrano* cheese produced in *Santa Catarina* state, two of the studies in the reviewed literature [15,41] demonstrated that 60 days of ripening may not be enough to meet the legal parameters since *L. monocytogenes* was present at 35 [41] and 60 days [15] of ripening. However, for *Serrano* cheese produced in *Rio Grande do Sul* state, Souza et al. [68], Pretto et al. [48], and Ströher et al. [49] indicated a minimum ripening time ranging from 30 to 33 days, which is a much shorter ripening period that can still reach acceptable microbiological levels than those required by Normative Instruction SEAGRI N° 7 [63]. It is important to highlight that Ströher et al. [49] demonstrated that the refrigerated temperature (5 °C) determined by IN 7/2014 is not adequate for ripening this type of cheese.

Regarding *Colonial* cheese produced in *Santa Catarina* state, Degenhardt et al. [47] observed that thermotolerant coliform were the most resistant throughout the cheese ripening. Although other bacterial pathogen counts reached acceptable levels earlier in the process, some of the samples only started to present acceptable levels for thermotolerant coliform bacteria after 28 days of ripening.

No other studies were found regarding the minimum ripening time to ensure cheese safety of other Brazilian artisanal cheese types. Therefore, there is no consensus in the scientific literature on the minimum required ripening time for each artisanal cheese type in Brazil. It is noteworthy that a large portion of the studies that were found assessed the cheeses for 60 days at most, thus making it impossible to ascertain the behavior of bacteria after this period. That is likely because longer ripening periods may lead to physical–chemical, structural, and flavor changes, as well as lessened consumer acceptance of the product [65]. Nevertheless, studies conducted in other countries demonstrate that, in certain situations, even 60 days of ripening may not suffice for eliminating certain pathogens [69,70,71,72].

Given that the reviewed literature lacks a standardized methodology to ensure sufficient ripening time for producing safe cheeses, Table 2 presents the diverse ripening conditions observed in the evaluated studies to facilitate a more comprehensive interpretation of the gathered data.

It is essential to emphasize that none of these studies assessed the presence of zoonotic pathogens that are challenging to detect, such as *Brucella* sp. [73] and *Mycobacterium* sp. [74], nor did these studies investigate their population behavior throughout the ripening process. They were not considered potential hazards since it was assumed that the herd was free of these pathogens when the artisanal cheeses were produced [75]. However, this matter has been a point of contention because only producers who have their facilities registered at official inspection services provide this guarantee, despite the fact that the majority of producers are not registered [12].

During ripening, whether artisanal or non-artisanal, cheese undergoes profound physical, chemical, and microbiological changes, and these are influenced by environmental and enzymatic factors. In artisanal cheeses, organic acids, primarily produced by the milk’s natural bacteria, also play a significant role in the development of the unique characteristics of different types of cheese that occurs throughout ripening [76].

Seasonal variation also impacts bacteria in artisanal cheeses by affecting the cheese’s moisture. During the wet season, moisture tends to be higher; thus, the cheese population of the total and thermotolerant coliforms is also greater [28]. Moreover, higher temperatures favor the growth and metabolism of lactic acid bacteria (LAB), thus making it a positive factor in cheese ripening [14].

The LAB present in milk and cheese are more complex than those in the commercial starter culture used in cheese made with pasteurized milk. These bacteria are responsible for essential reactions in cheese making, particularly the lipolysis and proteolysis that occur during ripening. This results in compounds related to desirable flavor and texture features, which are unique to each kind of artisanal cheese [77,78,79,80,81,82].

During the ripening process, competition occurs between secondary microbiota and the LAB, leading to lactic acid production, a reduction in sugar content, and a decrease in the redox potential of the cheese. The production of substances that inhibit the presence of pathogenic bacteria also occurs [83,84]. These substances include, but are not limited to, bacteriocins, which are peptides with antibacterial activity against species phylogenetically related to those that produce them [85]. By binding to the cellular surface or entering the cellular space, bacteriocins create pores in the cell membrane and inhibit the production of peptidoglycan [86].

Since various studies reported an array of LAB with functional, technological, and antimicrobial activities, these bacteria may play an important role in improving the overall quality and microbiological safety of artisanal cheeses [83,87,88].

The aforementioned findings underscore the existing knowledge gaps regarding cheese contamination with foodborne pathogens (zoonotic and non-zoonotic) and their inactivation kinetics throughout the ripening process. Additionally, contamination after processing, as well as during transportation, storage, and retail, should be prevented by instructing everyone involved in the process. The safety of cheese must be ensured throughout all production and marketing chains.

## 2. Conclusions

Analysis of the literature revealed that the ripening conditions of Brazilian artisanal cheeses made with raw milk might not be sufficient for reducing hazardous bacterial levels to acceptable limits and ensuring product safety for all cheese producers. Ingredient quality control, which is preceded by cattle health monitoring, good hygienic milking, and good manufacturing practices, are of utmost importance in keeping indicator organisms low and preventing pathogens from entering the production system. Additionally, care must be taken during the storage, transportation, and display stages for sale to avoid post-processing contamination of the cheese.

While cases of Brazilian artisanal cheese contamination by pathogenic bacteria with significant public health implications, such as *Coxiella burnetii* and *Brucella* spp., are rare, some studies have indicated that these organisms can withstand the ripening period. There is a lack of studies regarding the ripening period, temperature, and kinetics of the inactivation of zoonotic foodborne pathogens to ensure that the established ripening period and temperature for different types of artisanal cheese produced in the country are effective for inactivation.

It was noted that there are no patterns in the protocols of studies, with each author defining their own protocol. Considering that artisanal cheeses have unique microbiota compared to cheeses made from pasteurized milk, assessments of the microbiological safety for this cheese must be more specific. In addition to indicator bacteria, studies should also consider some of the zoonotic pathogens that are more common in bovine herds, as well as the extrinsic and intrinsic factors related to ripening.

To prevent such contamination in the artisanal cheese, it is important to provide producers with appropriate training and technical support that specifically address these types of pathogens. Furthermore, surveillance and inspection measures should be expanded to encompass these bacteria within their standards. By doing so, these pathogens can effectively be eliminated from the production chain, thus preventing contamination of the final product and ensuring consumer safety.

## Figures and Tables

**Table 1:** Non-compliant samples of the main ripened Brazilian artisanal cheeses obtained by considering the literature available at PubMed, SciELO, Science Direct, Scopus, and the Web of Science between September 2021 and April 2024.

Cheese (Place)	Ripening Conditions ¹	Samples (n)	Microorganisms	Non-Compliant Samples (%) ^2,3^	Reference
*Serro* (MG)	3 to 8 days	53	CB ^6^	9.43	[32]
*Serro* (MG)	30 days refrigerated (10 °C)	40	TC	80.00	[33]
			TTC	60.00	
			CPS	82.50	
*Serro* (MG)	17 to 22 days	53	CPS	75.50	[10]
*Canastra* (MG)	22 days	78	CPS	42.00	[12]
			TC	19.00	
			EC	18.00	
			LM	1.00 ^4^	
QMA (MG)	NI	100	SL	1.00	[34]
			LM	3.00	
			EC	10.00	
			SA	32.00	
QMA (MG)	NI	23	TC	100.00	[35]
			TTC	100.00	
			CPS	100.00	
			SL ^5^	2.50	
QMA (MG)	NI	19	EB	68.42	[36]
			CPS	68.42	
Araxá (MG)	14 to 22 days	99	TC	23.20	[37]
			EC	17.20	
*Campo das Vertentes* (MG)	22 days	24	TC	15.00	[37]
			EC	12.50	
*Canastra* (MG)	14 to 22 days	337	TC	14.80	[37]
			EC	12.50	
			LM	0.89	
*Cerrado* (MG)	22 days	206	TC	27.20	[37]
			EC	22.30	
			LM	0.48	
*Serra do Salitre* (MG)	14 to 22 days	40	TC	15.00	[37]
			EC	12.50	
*Serro* (MG)	17 days	664	TC	35.40	[37]
			EC	17.00	
			LM	0.45	
			SL	0.15	
*Triângulo Mineiro* (MG)	22 days	20	EC	5.00	[37]
*Araxá* (MG)	22 days	56	SA	42.90	[38]
*Campo das Vertentes* (MG)		54		31.50	
*Cerrado* (MG)		54		42.60	
*Canastra* (MG)		48		35.40	
*Serro* (MG)	17 days	50	SA	30.00	[38]
*Cerrado* (MG)	2 to 3 days	87	CB ^6^	4.60	[39]
*Cerrado* (MG)	NI	15	CPS	46.66	[40]
			SL	6.66	
*Colonial* (SC)	NI	12	CPS	100.00	[13]
			TC	82.00	
			TTC	66.70	
*Colonial* (NI)	30 days	55	SA	60.00	[38]
*Serrano* (SC)	15 to 60 days	108	TC	34.26	[15]
			EC	36.11	
			SA	33.33	
			LM	2.77	
*Serrano* (SC)	14 to 35 days at room temperature	76	TC	67.10	[41]
			EC	48.68	
			CPS	32.89	
			LM	5.26	
*Serrano* (RS)	NI	20	SL	10.00	[42]
			CPS	60.00	
			TTC	55.00	
			TC	100.00	
*Serrano* (NI)	15 to 30 days	48	SA	62.50	[38]

^1^ The ripening temperature of the [10,15,32,37,38,39] studies was not included in the studies. ^2^ According to Brazilian Federal Ordinance n° 146 [29]. ^3^ Plate count methodology except for coliforms that used the MPN methodology. ^4^ PCR methodology. ^5^ On the first day of ripening. CB: *Coxiella burnetiid*; TC: total coliforms; TTC: thermotolerant coliforms; EB: *Enterobacteriaceae*; EC: *Escherichia coli*; SL: *Salmonella*; LM: *Listeria monocytogenes*; CPS: coagulase-positive *Staphylococcus*; SA: *Staphylococcus aureus*; MG: *Minas Gerais* state; QMA: *Queijo Minas Artesanal*; RS: *Rio Grande do Sul* state; SC: *Santa Catarina* state; and NI: not informed. ^6^ This organism is not set as a requirement on the Brazilian artisanal cheese legislation but was included due to its importance as a resilient pathogen.

**Table 2:** Different ripening conditions of the evaluated cheeses according to several studies when considering the literature available at PubMed, SciELO, Science Direct, Scopus, and the Web of Science between September 2021 and April 2024.

Cheese	Local	Ripening Time	Ripening Conditions	Reference
*Canastra*	MG	64 days	Room temperature or refrigerated	[18]
			Rainy or dry season	
*Canastra*	MG	22 days *	Room temperature **	[12]
*Serra do Salitre*	MG	60 days	Room temperature	[67]
*Serro*	MG	64 days	Room temperature or refrigerated	[14]
			Rainy or dry season	
*Serro*	MG	60 days	Refrigerated	[28]
			Rainy or dry season	
*Serro*	MG	60 days	Room temperature	[66]
*Serrano*	SC	60 days	Not described	[14]
*Serrano*	SC	35 days	Room temperature	[41]
*Serrano*	RS	60 days	Room temperature	[68]
			Rainy or dry season	
*Serrano*	RS	60 days	Refrigerated	[48]

* It is important to note that the ripening time is now set at 14 days by the new legislation from *Minas Gerais* state. ** Cheese samples were collected directly from producers.

## Data Availability

No new data were created or analyzed in this study. Data sharing is not applicable to this article.
